# Increase in newly diagnosed type 1 diabetes and serological evidence of recent SARS-CoV-2 infection: Is there a connection?

**DOI:** 10.3389/fmed.2022.927099

**Published:** 2022-07-28

**Authors:** Marco Denina, Michela Trada, Davide Tinti, Elisa Funiciello, Chiara Novara, Martina Moretto, Sergio Rosati, Silvia Garazzino, Claudia Bondone, Luisa De Sanctis

**Affiliations:** ^1^Department of Pediatric Emergency, A.O.U. Città della Salute e della Scienza di Torino, Turin, Italy; ^2^Infectious Diseases Unit, Department of Pediatrics, A.O.U. Città della Salute e della Scienza di Torino, University of Turin, Turin, Italy; ^3^Department of Pediatrics, Center of Pediatric Diabetology, A.O.U. Città della Salute e della Scienza di Torino, University of Turin, Turin, Italy; ^4^Department of Pediatrics and Public Health, A.O.U. Città della Salute e della Scienza di Torino, University of Turin, Turin, Italy; ^5^Department of Veterinary Science, University of Turin, Turin, Italy

**Keywords:** children, SARS-CoV-2, type 1 diabetes, diabetic ketoacidosis, COVID-19, SARS-CoV-2 antibodies

## Abstract

Several studies have investigated the correlation between the COVID-19 pandemic and the onset of type 1 diabetes (T1D) in children, reporting an increased incidence of T1D and severe diabetic ketoacidosis (DKA). This study aimed to investigate the infection by SARS-CoV-2 in children with newly-diagnosed T1D to explore a possible link between SARS-CoV-2 infection, T1D and DKA. Thirty-nine children with a T1D new onset between October 15, 2020, and April 15, 2021, were enrolled. SARS-CoV-2 infection was investigated through a polymerase chain reaction on the nasal swab, dosage of specific antibodies, and an anamnestic question form. Nine (23%) of them had antibodies directed toward SARS-CoV-2, and five (12%) had a history of recent SARS-CoV-2 infection in themselves or in their family. No molecular swabs were positive. Compared to the general pediatric population, the overall incidence of COVID-19 was 5.6 times higher in the T1D patients' group (*p* < 0.00001). Referring only to the cases in the metropolitan area, we find a net increase in the incidence of T1D compared to the 5 years preceding our study, by 50% compared to the same months in 2016/2017 and 2017/2018, by 69% compared to 2018/2019 and by 77% compared to 2019/2020. The same trend was observed regarding DKA cases. The attributable risk of the pandemic cohort compared to the previous year is 44%. The abnormal disproportion of SARS-CoV-2 infection between children with T1D and the pediatric reference population, with a ratio of 5.6, appears to support the causative role of SARS-CoV-2 in triggering the immune response underlying diabetes, as often described for other viral infections. The difficulty accessing care services during the pandemic, with a consequent diagnosis delay, does not justify the increase in observed T1D cases, which could to be directly linked to the pandemic. The acceleration of the immune process provoked by SARS-CoV-2 may play a suggestive role in the development of T1D with DKA. Multicenter studies are needed to deepen and fully understand the pathophysiological link between SARS-CoV-2 and the onset of T1D in children.

## Introduction

Since the beginning of the COVID-19 outbreak, several studies have investigated the correlation between the pandemic and the onset of type 1 diabetes (T1D) in children, reporting an increased incidence of T1D and severe diabetic ketoacidosis (DKA) (1–4). Moreover, an increased incidence of T1D was also noted in a big population in the US (5). Given the disease's autoimmune nature, exogenous triggers (such as viruses) have been linked to T1D development in the past years (6). During the COVID-19 pandemic, lockdown reduced the viral spreading of different viruses (7). Therefore, a possible link between SARS-CoV-2 infection and the development of diabetes and DKA was hypothesized.

## Methods

The present study aimed to investigate the infection by SARS-CoV-2 in children with newly-diagnosed T1D during the second wave of the COVID-19 pandemic to explore a possible link between SARS-CoV-2 infection, T1D and DKA. The study includes all the 39 patients who presented to the emergency department for T1D new onset between October 15, 2020, and April 15, 2021, aged between 0 and 14 years, in the Turin area (Piedmont, Italy). We investigated an ongoing or recent SARS-CoV-2 infection through a polymerase chain reaction of nasal swab and dosage of SARS-CoV-2 specific antibodies in each patient enrolled in the study at admission. Due to the absence of seroprevalence data in our study population, we used nasal swab molecular analysis as a reference. In addition, each patient was subjected to a question form to evaluate medical history and, whenever applicable, to identify and to date SARS-CoV-2 infection. Demographic data, clinical presentation, and T1D characteristics at blood sample collection were also registered. Written informed consent was obtained from the parents of patients, and the Institutional Ethical Committee approved the study (protocol number 0000168). Molecular tests were performed with Simplexa™ COVID-19 Direct kit (Diasorin, Saluggia, Italy); the PCR assay targets two different regions of the SARS-CoV-2 genome, ORF1 and S (spike) genes. We investigated the presence of anti-SARS-CoV-2 antibodies using an ELISA assay (In3diagnostic Eradikit COVID-19, Turin, Italy) with a reported sensibility of 96% (8). Data were statistically analyzed using SPSS Statistic v23 (IBM, Armonk, NY). Continuous variables with non-normal distribution were expressed as medians (interquartile ranges), and categorical variables were expressed as numbers (percentages) and were compared with the χ^2^ test or Fisher exact test. A 2-sided *p*-value < 0.05 was considered statistically significant.

## Results

During the study period, 39 newly diagnosed T1D pediatric patients were enrolled, 20 (51.2%) males and 19 (48.8%) females, with a median age of 8.5 years (IQR 5.6–11.2 yrs.). In 9 (23%), antibodies directed toward SARS-CoV-2 were identified. Five patients had a history of recent SARS-CoV-2 infection in themselves or in their family, but in the other 4 cases, no clinical or anamnestic infection evidence since March 2020 was disclosed. No molecular swabs were positive for SARS-CoV-2 collected upon admission of the patients to the emergency department.

Compared to the previous period, the incidence of COVID-19 (resulting from a positive nasal swab) in the Piedmont pediatric population rose from 0.3 (on 15/10/2020) to 4.1% (on 15/04/2021) (9), resulting in an overall incidence at the end of the study 5.6 times higher in the T1D patient's group (*p* < 0.00000001).

Referring only to the cases in the area surrounding the hospital considering the variations in the pediatric population, we also analyzed the incidence of T1D in the 5 years preceding our study, finding a net increase (Table 1) in the cases which was statistically significant. In particular, the number of newly diagnosed T1D increased by 50% compared to the same months in 2016/2017 and 2017/2018, by 69% compared to 2018/2019 and by 77% compared to 2019/2020. The attributable risk to the pandemic in the 2020–2021 cohort compared to the previous year is 44%.

**Table 1:** Number of annual newly diagnosed T1D and DKA from 2016 to 2022.

**Period**	**No of new onset T1D**	**Percentage increase in the study period**	**p**	**No of T1D presented with DKA**	**Percentage increase in the study period**	**p**	**Turin pediatric population—no**
October 2016–April 2017	26	50.0%	**0.049**	8	100.0%	0.064	110,325
October 2017–April 2018	26	50.0%	0.056	7	128.6%	**0.039**	108,911
October 2018–April 2019	23	69.6%	**0.022**	7	128.6%	**0.041**	107,974
October 2019–April 2020	22	77.3%	**0.024**	11	45.5%	0.31	103,291
October 2020–April 2021	39			16			101,136
October 2021–March 2022 *(preliminary data)*	39			14			

Finally, we analyzed the number of T1D presented with DKA in 2020/2021, comparing them with the previous year. As shown in Table 1, the increase in DKA cases was clear and significant, especially compared to 2017–2018 and 2018–2019. Interestingly, preliminary data on the current season, from October 2021 to March 2022, overlap with those of our study period (Table 1).

## Discussion

A link between common viral infections and T1D in children is widely reported, acting through several mechanisms (10–12). Several viral infections are associated withT1D (13), with enterovirus being one of the most commonly associated, and Enteroviral major capsid protein VP1 and RNA have been detected in islets from people with recent-onset type 1 diabetes (14).

First, viruses can cause direct damage to pancreatic B-cells, resulting in the release of specific antigens that can act as a target for the immune response. Second, homologies between viral epitopes and autoantigens may stimulate the production of cross-reactive antibodies. Third, the immune activation accompanying a viral infection may reinforce an auto-immune response in individuals genetically predisposed (15), and viral infection may act as an accelerator in the immunological process which leads to T1D (16).

These mechanisms are common in all viral infections and are also described in SARS-CoV-2 infection: recent evidence suggests that SARS-CoV-2 can infect and replicate into pancreatic Beta cells and alter the pathway of insulin synthesis (17). Nevertheless, according to current knowledge, *in-vitro* models cannot fully explain the pathophysiological link between SARS-CoV-2 and T1D. However, in the last 2 years, epidemiological and clinical studies have extensively described the connection between COVID-19, newly diagnosed T1D, and DKA increase at clinical presentation (1–3).

We observed that 23% of T1D patients had a positive SARS-CoV-2 serology while only 4% of the pediatric population experienced COVID-19 in the same time period. Children were not vaccinated against SARS-CoV-2 at the moment of this observational study. We do not have epidemiological data about other viruses, but we observed less hospital admittance for any other cause during the study period. This abnormal disproportion between our children with T1D and the pediatric reference population, with a ratio of 5.6, appears to support the causative role of SARS-CoV-2 in triggering the immune response underlying diabetes, as often described for other viral infections. Additionally, we identified a 44% increased risk attributable to the pandemic years compared to previous years. Most of the patients investigated had precise exposure to the virus in the past month. As for patients with no known history of COVID-19, the higher antibody anti-SARS-CoV-2 title suggests that they developed a paucisymptomatic disease, followed by some immune response that caused or emerged T1D. While it seems unlikely that patients developed COVID-19 previously (the first lockdown, from March 9, 2020, to May 3, 2020, was extremely rigid), it is possible that the immune process leading to T1D could be ongoing at the moment of infection, and SARS-CoV-2 only accelerated T1D onset.

Although there are minor discrepancies based on the study's characteristics and the reference populations, the COVID-19 pandemic was characterized by an increase in the absolute number of cases of T1D onset and the severity (DKA) with which they occurred (18, 19). One of the reasons for understanding the DKA rise is the difficulty accessing care services during the pandemic during lockdown, with a consequent diagnosis delay, as demonstrated from an Italian survey which observed less cases of diabetes during first 2 months of pandemic but more DKA (20). Although partial, this explanation does not justify the increase in observed T1D cases, which appear to be directly linked to the pandemic. In this scenario, the acceleration of the immune process provoked by SARS-CoV-2 may seem more explicative in the development of T1D with DKA.

Different from Tittel et al. (1), which found a nominal increase in T1D rates among males but a nominal decrease in rates among females, in our study, we describe a percentage increase in the prevalence of females in the composition of the cohort of diabetic patients. This increase is not statistically significant, probably due to one of the major limitations of our study, namely the small size of the study population.

A major study limitation is the lack of seroprevalence data in the reference population, which is not available since no mass screening was done at the time of the study. If we use data from a study carried out in children referring to the hospital for any reason in an area with similar characteristics, the seroprevalence of SARS-CoV-2 antibodies was 9.5% (21). Given that, we can consider that value as a reference to evaluate the prevalence of Sar-Cov-2 infection and, although data are not comparable in a strict statistical modality, patients with T1D would have more than a double seroprevalence for SARS-CoV-2 as other patients, supporting the thesis proposed so far. Additionally, as reported in other studies, we have identified a significant increase in the absolute number of T1D cases compared to previous years. The trend also persists when we analyze preliminary data about the ongoing winter season. Unfortunately, we have no serological data about this season, but the trend is still significant. Therefore, the rise of T1D during the two pandemic years compared with the previous 5 years cannot be split from the circulation of SARS-CoV-2 and appear to be attributable to the viral infection, as largely reported in the literature.

## Conclusion

SARS-CoV-2 infection is potentially linked to a rise in newly diagnosed T1D and DKA in our population. The SARS-CoV-2 virus can act as a trigger or accelerator in T1D development. Multicenter studies with precise dating of viral infection are needed to deepen and fully understand the pathophysiological link between SARS-CoV-2 and the onset of T1D in children.

## Data availability statement

The raw data supporting the conclusions of this article will be made available by the authors, without undue reservation.

## Ethics statement

The studies involving human participants were reviewed and approved by A.O.U. City of Health and Science of Turin—A.O. Mauritian Order—A.S.L. City of Turin. Written informed consent to participate in this study was provided by the participants' legal guardian/next of kin.

## Author contributions

MD and MT conceptualized and designed the study, drafted the initial manuscript, and reviewed and revised the manuscript. LD and DT designed the data collection instruments, coordinated and supervised data collection, and critically reviewed the manuscript. CN, MM, and EF collected data and reviewed the manuscript. SG and CB reviewed and revised the manuscript. All authors approved the final manuscript as submitted and agreed to be accountable for all aspects of the work.

## Conflict of interest

The authors declare that the research was conducted in the absence of any commercial or financial relationships that could be construed as a potential conflict of interest.

## Publisher's note

All claims expressed in this article are solely those of the authors and do not necessarily represent those of their affiliated organizations, or those of the publisher, the editors and the reviewers. Any product that may be evaluated in this article, or claim that may be made by its manufacturer, is not guaranteed or endorsed by the publisher.
